# Secretome analysis reveals upregulated granzyme B in human androgen-repressed prostate cancer cells with mesenchymal and invasive phenotype

**DOI:** 10.1371/journal.pone.0237222

**Published:** 2020-08-07

**Authors:** Mayassa J. Bou-Dargham, Qing-Xiang Amy Sang

**Affiliations:** 1 Department of Chemistry and Biochemistry, Florida State University, Tallahassee, Florida, United States of America; 2 Institute of Molecular Biophysics, Florida State University, Tallahassee, Florida, United States of America; MAHSA University, Malaysia, MALAYSIA

## Abstract

Epithelial-mesenchymal transition (EMT) is a critical early step in cancer metastasis and a complex process that involves multiple factors. In this study, we used proteomics approaches to investigate the secreted proteins (secretome) of paired human androgen-repressed prostate cancer (ARCaP) cell lines, representing the epithelial (ARCaP-E) and mesenchymal (ARCaP-M) phenotypes. Liquid chromatography-tandem mass spectrometry (LC-MS/MS) analyses showed high levels of proteins involved in bone remodeling and extracellular matrix degradation in the ARCaP-M cells, consistent with the bone metastasis phenotype. Furthermore, LC-MS/MS showed a significantly higher level of the serine protease granzyme B (GZMB) in ARCaP-M conditioned media (CM) compared to that of ARCaP-E. Using quantitative reverse-transcriptase polymerase chain reaction (qRT-PCR) to detect mRNA and Western blot to detect protein expression, we further demonstrated that the GZMB gene was expressed by ARCaP-M and the protein was secreted extracellularly. ARCaP-M cells with GZMB gene knockdown using small interfering RNA (siRNA) have markedly reduced invasiveness as demonstrated by the *Matrigel* invasion assay in comparison with the scrambled siRNA negative control. This study reports that GZMB secretion by mesenchymal-like androgen-repressed human prostate cancer cells promotes invasion, suggesting a possible extracellular role for GZMB in addition to its classic role in immune cell-mediated cytotoxicity.

## Introduction

Prostate cancer is the most common cancer among men in the United States, aside from non-melanoma skin cancer, according to the Centers for Disease Control and Prevention (CDC). Two-thirds of cancer-related deaths in the US involve bone metastasis and prostate tumors in particular are prone to disseminate to the bone [1]. Despite the recent advances in clinical trials, cancer metastasis still accounts for the majority of cancer deaths and metastatic prostate cancer remains an incurable disease [2–4].

Metastasis is a multistep process that involves genetic and phenotypic changes that allow cancer cells to leave their primary site and colonize secondary sites. Cancer cells undergo epithelial-mesenchymal transition (EMT) to overcome apoptosis and induce anchorage-independent growth. Thus, they lose their intercellular connections, intravasate the local environment to the bloodstream, then extravasate to secondary tissues and undergo a mesenchymal-epithelial transition (MET) [5,6]. EMT is further aided by cancer cells’ decreased expression of epithelial markers such as E-cadherin and cytokeratins and increased expression of mesenchymal markers such as N-cadherin, vimentin, and fibronectin [7,8]. EMT is thus a critical step for the initiation and transformation of benign cancer to metastatic [5].

Prostate cancer is commonly treated with castration and androgen deprivation therapy (ADT) when it is androgen-dependent [9]. The cancer may progress to become androgen-independent, also known as castrate-resistant [10], with certain clones progressing into the androgen-repressed phenotype [11]. Castration-resistant prostate cancer may respond to secondary hormone therapy manipulations such as antiandrogen withdrawal and other androgen inhibitors [9,11,12]. Yet, androgen-repressed prostate cancer is highly invasive and metastatic [11]. Thus, managing metastasis by investigating EMT in the androgen-repressed subtype is an unmet need in prostate cancer.

Androgen-repressed prostate cancer (ARCaP) cells were isolated from the ascites fluid of a man diagnosed with metastatic carcinoma of the prostate [11,13]. Epithelial like ARCaP (ARCaP-E) cells were induced to undergo EMT by exposing them to soluble factors or bone microenvironment [14]. The resulting ARCaP-M had a 100% incidence of bone metastasis further validating the importance of EMT in metastasis [14]. Thus, the ARCaP-E and ARCaP-M cell lines represent a good model for studying EMT and identifying potential therapeutic targets to manage cancer progression. Proteomics and phosphoproteomics studies have been conducted on the ARCaP-E/ARCaP-M cell line model to study differentially expressed proteins [15,16]. However, differentially secreted proteins have not been investigated before.

Granzyme B (GZMB) is a serine protease traditionally known for being expressed by cytotoxic T lymphocytes (CTL) and natural killer (NK) cells to induce apoptosis in tumors and virally transfected cells through caspase-activating pathways once they reach the target cell’s cytoplasm [17,18]. GZMB is currently used as an indicator of CTL activation in tumors, and its positive immunostaining is associated with a favorable clinical outcome in a variety of cancers [19]. GZMB gains access to a target cell’s cytoplasm by perforin-mediated pore formation in the cell’s plasma membrane. More recent work has shown that GZMB can be secreted by other non-immune cells, and cleavage sites were identified not only in intracellular proteins, but also in extracellular matrix components, cell surface receptors, cytokines, and growth factors [17,20–23]. Despite the favorable outcome associated with GZMB expression in tumors, its expression in some cases was associated with poor prognosis, resistance to therapy, and advanced disease stage [19,24–28]. Given that GZMB requires perforin or more specific receptors to enter a target cell’s cytoplasm and induce a cytotoxic effect by CTLs and NK cells, its secretion by non-immune cells without perforin suggests a role in the degradation of ECM components. As an extracellular protein, GZMB can degrade ECM components such as vitronectin, fibronectin, and laminin in addition to the ECM structural proteoglycan, aggrecan [23]. The expression of GZMB by cancer cells has only been reported in bladder and pancreatic cancers where extracellular GZMB was found to promote their invasion *in vivo* [29,30].

In this study, we aim to further the understanding of EMT in androgen-repressed prostate cancer by identifying differentially secreted proteins between the ARCaP-E and ARCaP-M cell lines. We showed that the secreted proteins in ARCaP-M had a significant enrichment in proteolysis, ECM disassembly, and regulation of osteoblast differentiation and ossification processes. For the first time in human prostate cancer, we identify GZMB secretion by ARCaP-M and its potential role in increasing cancer invasion.

## Results

### Upregulated proteolysis and bone differentiation in ARCaP-M

Significantly enriched processes in ARCaP-M secretome were involved in proteolysis and ECM disassembly (S1 Table). The upregulated ARCaP-M proteins involved in these processes include the aminopeptidases aminopeptidase N (ANPEP) and leucyl-cystinyl aminopeptidase (LNPEP) (6.8 and 5.7-fold, respectively), granzyme B (GZMB; 800-fold), and matrix metalloproteinase 1 (MMP1; 2.4-fold) (Table 1, S2 Table).

**Table 1:** Upregulated proteins in ARCaP-M conditioned media compared to ARCaP-E.

Gene	Gene name	P-adjusted	Fold Change
**GZMB**	Granzyme B	<0.001	800
**HSPG2**	Basement membrane-specific heparan sulfate proteoglycan core protein	<0.001	24
**LRP8**	Low-density lipoprotein receptor-related protein 8	0.028	11
**S100A13**	Protein S100-A13	0.042	10
**SERPINA6**	Serpin Family A Member 6/Corticosteroid-binding globulin	0.002	8.1
**ANPEP**	Aminopeptidase N	<0.001	6.8
**LNPEP**	Leucyl-cystinyl aminopeptidase	0.005	6.4
**SRGN**	Serglycin	0.007	6.2
**L1CAM**	Neural cell adhesion molecule L1	0.011	5.7
**CYR61**	Protein CYR61	0.020	4.3
**CTSL**	Cathepsin L1	<0.001	4
**LDLR**	Low-density lipoprotein receptor	0.006	4
**PCDH1**	Protocadherin-1	0.011	4
**TFRC**	Transferrin receptor protein 1	0.028	3.8
**PNP**	Purine nucleoside phosphorylase	0.048	3.6
**NPNT**	Nephronectin	0.003	2.7
**ALCAM**	CD166 antigen	0.002	2.6
**SDC4**	Syndecan-4	0.044	2.6
**MMP1**	Interstitial collagenase	0.005	2.4
**PSMA1**	Proteasome subunit alpha type-1	0.011	2

Fold Change represents the fold change of ARCaP-M/ARCaP-E.

Other enriched processes in ARCaP-M included the regulation of ossification and osteoblast differentiation. This indicates that upon transitioning to mesenchymal-like, ARCaPs secrete molecules that aid in metastasis and regulate bone differentiation. This is especially important since the bone is the primary metastatic site for ARCaP-M [14].

Granzyme B (GZMB) and basement membrane-specific heparan sulfate proteoglycan core protein (HSPG2) were the top differentially secreted proteins with 800 and 24-fold change in ARCaP-M compared to ARCaP-E. HSPG2 upregulation has been detected before in prostatectomy specimens. In addition, elevated levels of HSPG2 fragments were identified in the sera of patients with invasive prostate cancer and associated with increased secretion of MMPs [31].

### Upregulated cell survival and differentiation signaling in ARCaP-E

Among the significantly upregulated proteins in ARCaP-E conditioned media (CM) were neuronal cell adhesion molecule (NRCAM), pigment epithelial-derived factor (SERPINF1), and complement C1s subcomponent (C1S), which were only detected in ARCaP-E CM (S2 Table). NRCAM promotes cellular growth and differentiation [32], SERPINF1 inhibits angiogenesis in the prostate [33], and complements are peptidases that aid in cancer development [34,35].

The most significantly enriched processes in ARCaP-E were those that maintain survival by negatively regulating apoptosis and cell death. In addition, processes negatively regulating inflammatory response and cell migration, but positively regulating mesenchymal cell differentiation, were also significantly enriched (S1 Table). Thus, not only do ARCaP-E secretions help maintain viability and prevent inflammation, they also help keep them well differentiated.

### GZMB expression is significantly higher in ARCaP-M and its protein is detected extracellularly

**Fig 1:**
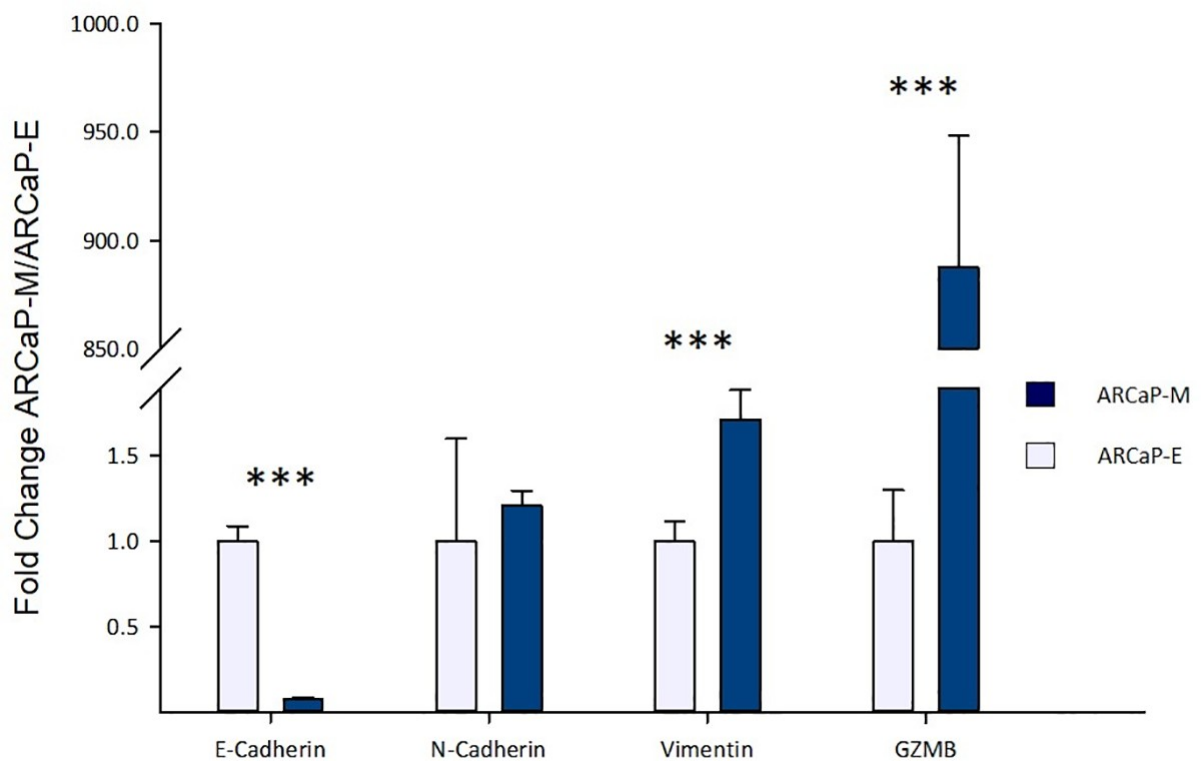
The relative gene expression of epithelial-mesenchymal transition (EMT) markers and GZMB in ARCaP-E (n = 3) and ARCaP-M (n = 3) using qRT-PCT. The samples are normalized against endogenous control (GAPDH). These results show approximately 900-fold higher gene expression of GZMB in ARCaP-M compared to ARCaP-E. Asterisks indicate statistical significance (***: p-value< 0.001).

**Fig 2:**
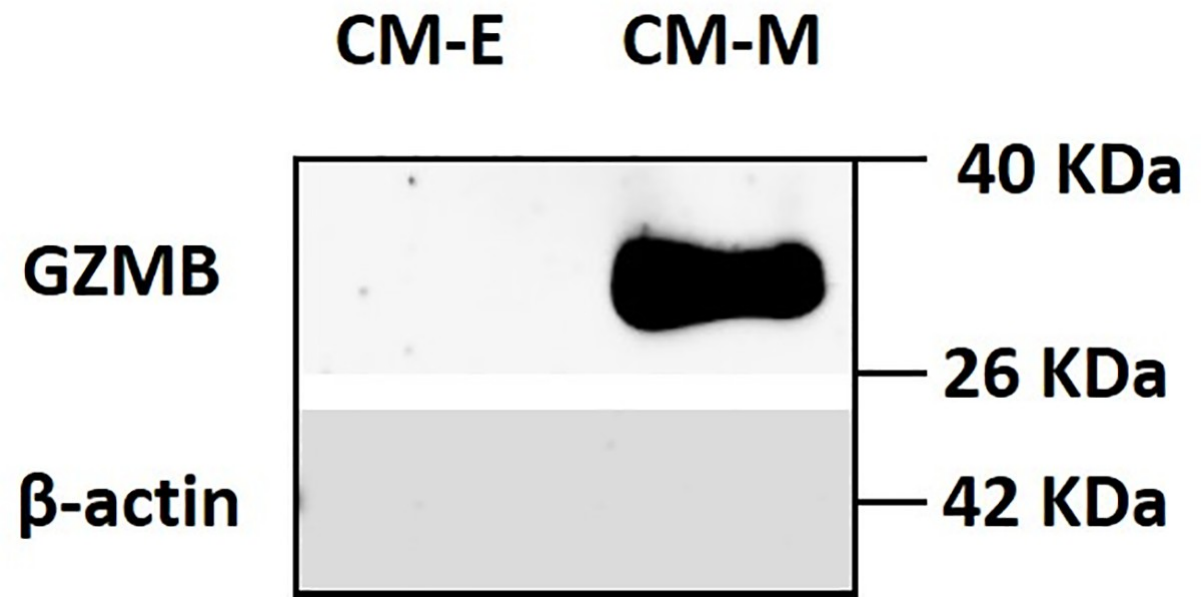
Western blot analysis to detect granzyme B (GZMB) protein expression in ARCaP-E and ARCaP-M conditioned media. The same amount of total protein was loaded for both ARCaP-E conditioned media (CM-E) and ARCaP-M conditioned media (CM-M). These results show the high level of GZMB secretion by ARCaP-M.

We checked the gene and protein expression of GZMB in ARCaP-M and ARCaP-E cell lines. The gene expression using qRT-PCR showed an 887-fold higher expression in ARCaP-M compared to ARCaP-E (Fig 1). Then the protein expression was checked in the CM of the two cell lines and a GZMB band was highly detected in the ARCaP-M CM (Fig 2). Thus, these results validate that GZMB is marginally expressed in ARCaP-E (mRNA) and highly secreted by ARCaP-M.

### GZMB aids in ARCaP-M cell invasion

**Fig 3:**
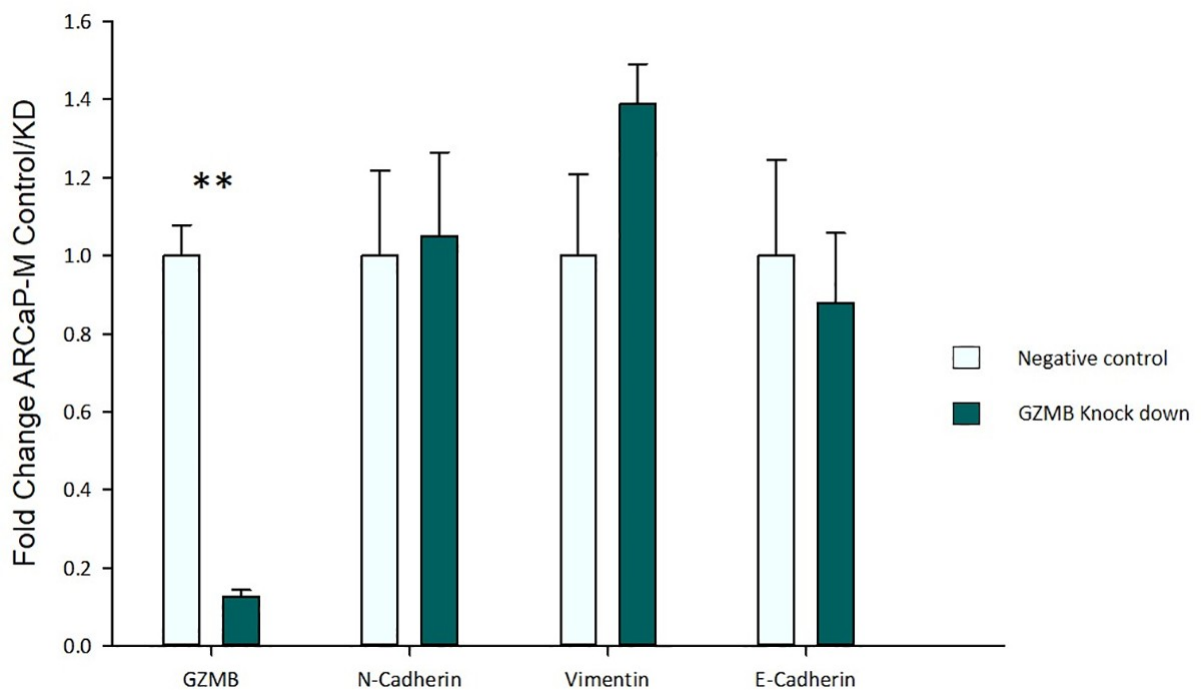
Relative gene expression of GZMB and EMT biomarkers after 48 hours of siRNA knockdown as measured by qRT-PCR. The relative expression is compared to the endogenous control (GAPDH). These results show that GZMB knockdown does not affect the expression of EMT biomarkers when compared to the negative control transfected with scrambled siRNA. Asterisks indicate statistical significance (**: p-value< 0.01).

To determine the function of GZMB, we knocked it down in the ARCaP-M cell line using siRNA. The knockdown efficiency was ~85% yet the expression levels of E-cadherin, N-cadherin, and vimentin were not affected (Fig 3).

**Fig 4:**
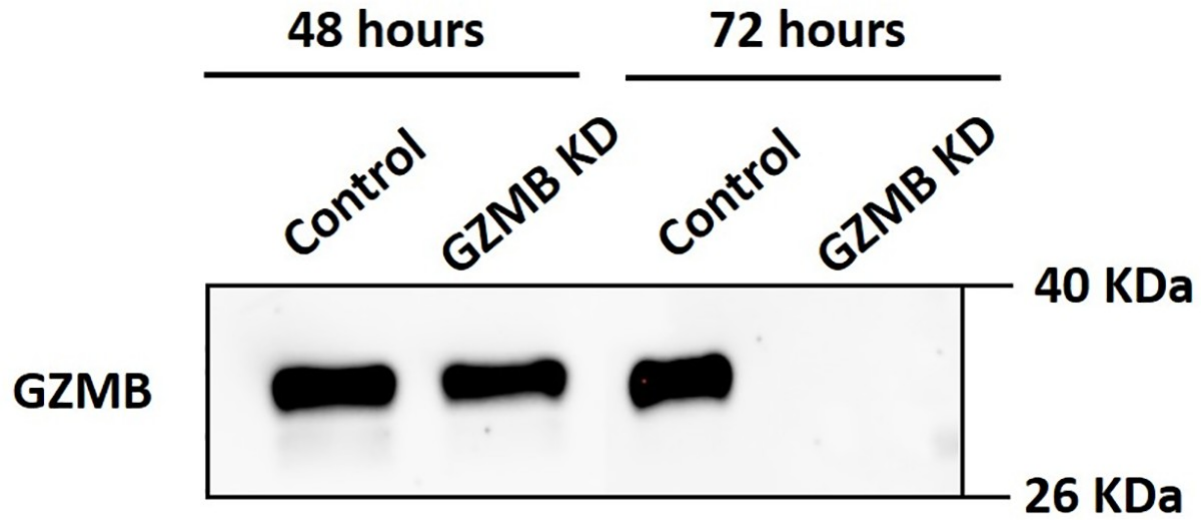
Western blot analysis to detect GZMB in conditioned media from ARCaP-M under negative scrambled siRNA control and GZMB siRNA knockdown (KD) after 48 and 72 hours. These results show the absence of GZMB in ARCaP-M 72 hours after GZMB knockdown and thus the 72-hour time point was selected for further investigations.

**Fig 5:**
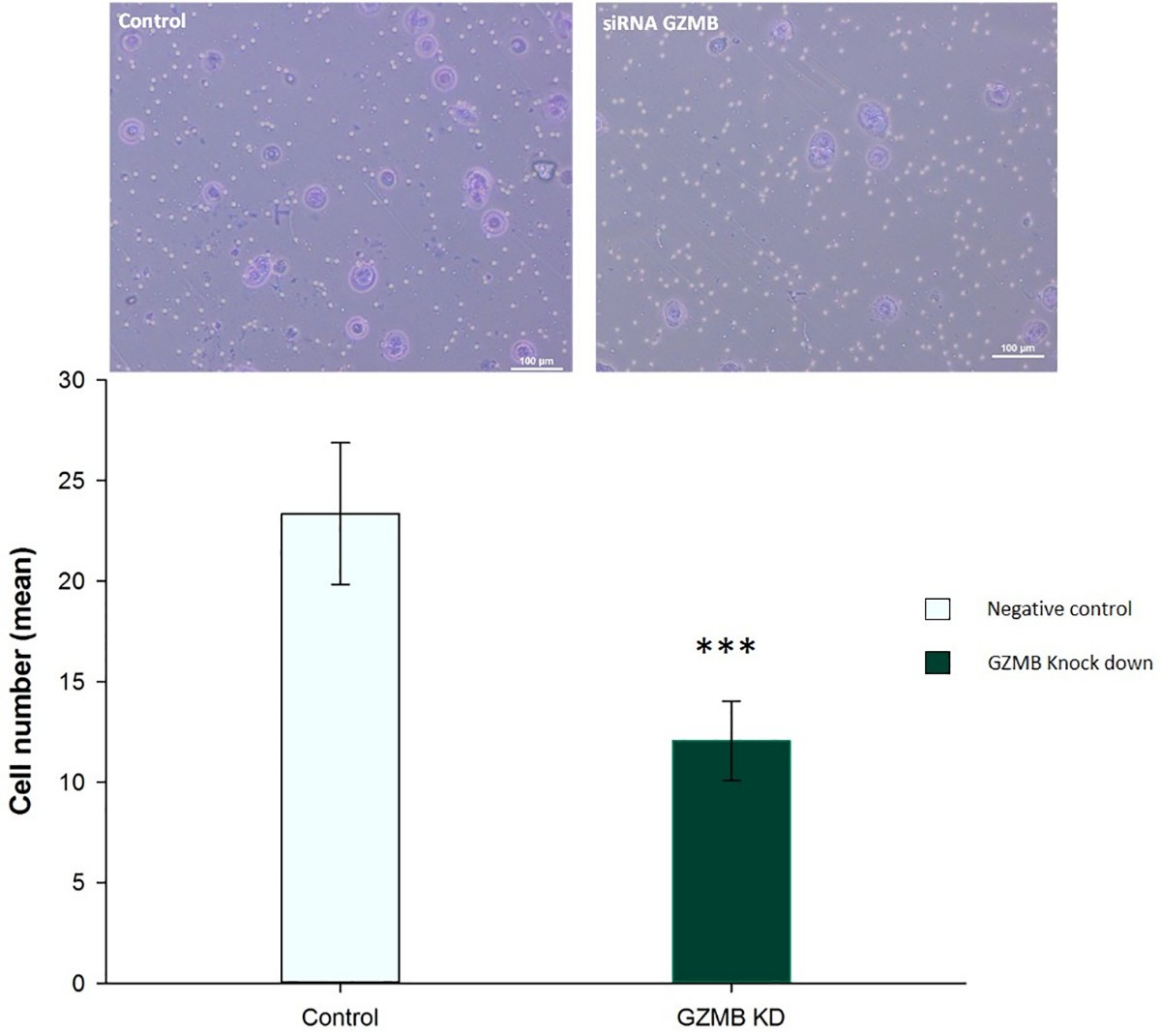
Invasion assay comparing ARCaP-M siRNA GZMB knockdown (KD) (n = 5) to ARCaP-M with scrambled negative control siRNA (n = 5). Pictures were taken using a Nikon microscope (10X magnification) and statistical analysis was done using a T-test. Asterisks indicate statistical significance (***: p-value <0.001).

Since GZMB is a proteolytic enzyme, we suspected it to have a role in invasion in ARCaP-M. Protein knockdown was checked in the CM collected at the 48-hour time point and the 72-hour time point. After the CM were removed at each time point, fresh media were added to the cell culture. The best efficiency at the protein level was determined at 72 hours and was thus used for further experiments (Fig 4). GZMB siRNA knockdown ARCaP-M cells were then checked for their invasion abilities using a Transwell invasion assay in comparison with a negative control transfection condition using a scrambled siRNA. After 72 hours of transfection, the knockdown ARCaP-M cells showed a significant decrease in their invasion abilities compared to the negative control (Fig 5), indicating that GZMB facilitates invasion.

## Discussion

While surgical removal, radiation therapy, and hormone therapies remain the most successful treatment options for early-stage prostate cancer, they tend to fail upon disease progression. Metastatic prostate cancer remains an incurable disease despite the current efforts and clinical cancer advancements [3,36]. Epithelial cancer cells’ transition to a mesenchymal-like phenotype (EMT) represents an early step of metastasis [37,38]. During EMT, epithelial cells lose cell-cell adhesion, gain mobility, and acquire the ability to degrade the basement membrane and other components of the extracellular matrix (ECM) [37,39–41].

Here we used the ARCaP-E/ARCaP-M cell line model, which represents a rare, late-stage prostate cancer, to investigate the molecular secretome signatures of the EMT process during the epithelial and mesenchymal states. By investigating the differentially secreted proteins, we identified significantly enriched processes that aid in cancer cell invasion, metastasis, and colonizing secondary sites.

Cancer cells tend to express markers that are bone-specific during, before, or after metastasis to the bone [42]. ARCaP-M cells induce bone remodeling by stimulating the differentiation of osteoclasts and osteoblasts to help them colonize the bone as a secondary site [43]. Only osteopontin was significantly higher in ARCaP-E but no GO processes were significantly upregulated for bone remodeling [43]. ARCaP-E CM contained molecules that maintain their survival, potentially to overcome anoikis [44], and others that induce mesenchymal cell differentiation. Thus, ARCaP-E cells maintain their epithelial phenotype potentially by inhibiting the de-differentiation processes. Given that EMT and MET are required for metastasis and colonizing secondary sites, respectively, the molecules involved in maintaining ARCaP-E differentiation may be the “brakes” that prevent their de-differentiation to ARCaP-M [5].

Although GZMB extracellular secretion by prostate cancer cells has not been previously reported, it was detected in our late-stage androgen-repressed prostate cancer, ARCaP-M, CM. Extracellular GZMB is not very common yet, some studies have reported a role in degrading ECM components such as vitronectin, fibronectin, and laminin in addition to the ECM structural proteoglycan, aggrecan [23]. Knockdown experiments using siRNA showed GZMB’s contribution to the ARCaP-M invasive phenotype by aiding in ECM degradation. Despite GZMB’s traditional role in inducing caspase-dependent apoptosis by CTL and its association with a good prognosis, several studies have shown other cancer-promoting functions [19,24–28]. Extracellular GZMB in pancreatic and bladder carcinoma cells was found to promote their invasion *in vivo* [29,30]. In addition, GZMB expression in colorectal cancer was associated with pathological tumor spreading and EMT [29,45].

This study highlights the important molecular pathways involved in EMT. Using the ARCaP-E/ARCaP-M cell line model, we were able to identify several differentially secreted proteins between the epithelial and mesenchymal phenotypes. For the first time in prostate cancer, GZMB was detected in ARCaP-M secretions and found to enhanced invasion. Inhibiting GZMB-mediated invasion was successfully accomplished in urothelial and colorectal carcinoma cells by docosahexaenoic acid (DHA) *in vitro* [30,45]. However, the effects of DHA on GZMB-induced invasion in human prostate cancer both *in vitro* and *in vivo*, remain to be investigated.

## Materials and methods

### Cell culture

ARCaP-E and ARCaP-M cell lines were generously provided by Drs. Haiyen E. Zhau and Leland W. K. Chung [14]. The cell lines were cultured in Dulbecco's Modified Eagle's Medium (Sigma Product # 5523) supplemented with 3.7 grams sodium bicarbonate in a humid incubator at 37°C and 5% CO2. For the secretome experiment, each cell line was cultured in 6 experimental replicates in phenol-free and serum-free media for 24 hours before the collection of conditioned media. For the knockdown experiment, the cells were cultured in antibiotics free media overnight and during transfection.

### Conditioned media and LC-MS/MS

Cells were incubated for 24 hours in phenol-free and serum-free media and viability was maintained at no less than 90%. Conditioned media from 6 experimental replicates of the ARCaP-E and ARCaP-M cultures were first centrifuged to get rid of any floating cells then passaged through a 0.2μm filter. The conditioned media were then concentrated using Amicon centrifugal filters Ultracel-3K (Merck Millipore Ltd Product # UFC900324). Protein concentration in concentrated CM was determined using bicinchoninic acid assay (BCA) and 60μg were loaded per well on a 12% SDS-polyacrylamide gel. The gel was then fixed and stained with Coomassie blue. Each lane was cut into 3 pieces and proteins were trypsinized in-gel. Peptides from each band were then run on LC-MS/MS Orbitrap in technical replicates. All MS/MS samples were analyzed using MS-Amanda Proteome Discoverer and Sequest (XCorr Only) (Thermo Fisher Scientific; Proteome Discoverer 2.2.0.388). MS-Amanda Proteome Discoverer and Sequest were set up to search human-specific databases (20180308HumanSwissprot.fasta) assuming the digestion enzyme is trypsin. MS-Amanda Proteome Discoverer and Sequest (XCorr Only) were searched with a fragment ion mass tolerance of 0.020 Da and a parent ion tolerance of 10.0 PPM.

Scaffold (version Scaffold_4.8.9, Proteome Software Inc., Portland, OR) was used to validate MS/MS-based peptide and protein identifications. Peptide identifications were accepted if they could be established at greater than 95.0% probability by the Peptide Prophet algorithm [46] with Scaffold delta-mass correction. Protein identifications were accepted if they could be established at greater than 99.9% probability and contained at least 2 identified peptides. Protein probabilities were assigned by the Protein Prophet algorithm [47]. Proteins that contained similar peptides and could not be differentiated based on MS/MS analysis alone were grouped to satisfy the principles of parsimony.

### Enrichment analysis

Significantly differentially expressed genes were checked for enriched biological processes using the String database (version 11.0) and the GO Term Mapper by Princeton University (https://go.princeton.edu/cgi-bin/GOTermMapper). Significant enrichment was considered at FDR < 0.05 for the String database and the GO slim terms were determined using GO Term Mapper based on the percent enrichment.

### Western blot

Western blot analysis was performed using cultured cells at 70–80% confluence. ARCaP-E and ARCaP-M triplicate wells were harvested using a lysis buffer (4% CHAPS, 8M Urea) supplemented with Halt protease and phosphatase inhibitor (ThermoScientific Product # 78443) and scraping. After collection, the cells were lysed by multiple freeze and thaw cycles and vortexing at 4°C for 1 hour. Cell lysates were then centrifuged for 15 minutes at 15,000 RPM at 4°C and the supernatant was collected. For the secreted proteins, media from three wells of ARCaP-E and 3 wells of ARCaP-M were collected and filtered as described above and in the following section. Proteins from cell lysates and conditioned media were quantified using BCA assay. In brief, 25μg of protein samples were loaded on 12% SDS-PAGE and run at 50V for 30 minutes then 100 volts. Proteins were transferred onto a nitrocellulose membrane (Thermo Fisher, 88018), blocked with 5% BSA for 2 hours, then incubated overnight with anti-GrB (GZMB, Santa Cruz, sc-73620) at 1:200 dilution, then with goat anti-mouse secondary antibody conjugated with horseradish peroxidase (Santa Cruz, sc-2005). Immune reactive bands were detected using the ChemiDoc MP™ Imaging System.

### Knockdown

siRNA targeting GZMB (Thermo Fisher catalog # 4392429, ID # s6389), sense sequence (5’ CUUAUGAUCUGGGAUCACAtt 3’) and antisense (5’ UCUGAUCCCAGAUCAUAAGat 3’) and a scrambled siRNA negative control (Catalog # 4390843) were used for the knockdown experiments. The transfection was done in triplicates using Lipofectamine 2000 and following the manufacturer protocol. Briefly, ARCaP-M cells were cultured such that they are between 30–50% confluent by the time of transfection. After 24, 48, and 72 hours the cells were harvested, and knockdown efficiency was checked using PCR and Western blot by comparing the GZMB siRNA knockdown to the negative control scrambled siRNA transfection. For the Western blot sample preparation, conditioned media were collected and fresh media were added to cell culture at 24-hour time point, 48-hour time point, and 72-hour time point, respectively. The conditioned media were concentrated as described previously.

### Quantitative reverse-transcriptase polymerase chain reaction (qRT-PCR)

RNA was extracted using the E.Z.N.A.^®^ total RNA kit (R6834) and following the manufacturer’s protocol. Then, the extracted RNA was cleaned to remove DNA contamination using the DNA-Free RNA Kit (R1013 by Zymo Research) followed by first-strand cDNA synthesis using the qScript™ cDNA supermix (Quanta catalog # 95048).

Real-time PCR was performed using the Applied Biosystems 7500 Fast with SYBR green PCR master mix (4309155) from Applied Biosystems. The PCR was run as follows: one cycle of 2 minutes at 50°C and 10 minutes at 95°C followed by 40 cycles of 15 seconds at 95°C, 30 seconds at 55°C and 30 seconds at 68°C. The oligonucleotide primers for PCR amplification were designed using primer blast and net primer and ordered from Eurofins Genomics. The sequences of the primers used are E-cadherin forward: 5’AGGGGTTAAGCACAACAGCA3’, reverse: 5’ACGACGTTAGCCTCGTTCTC3’; N-cadherin forward: 5’CAATTTGGGCTCAGA GGGAATA3’, reverse: 5’AGGCACATAAAATCCCAGTGCT3’; Vimentin forward: 5’ TCCGCAC ATTCGAGCAAAGA3’, reverse: 5’AACTTACAGCTGGGCCATCG3’; Granzyme B forward: 5’GA GCAAGGAGGAAACAACAGC3’, reverse: 5’TGATCTCCCCTGCATCTGCC3’.

### Invasion assay

After transfection, cells were serum-starved for 24 hours, trypsinized, and plated on Matrigel-coated Transwells in serum-free media while FBS-containing media were added to the bottom chamber to create a chemotactic gradient. Both GZMB siRNA transfected cells (5 wells) and the scrambled siRNA negative control (5 wells) were fixed with methanol, stained with crystal violet, and counted after 24 hours. Three random pictures were taken per Transwell using Nikon Eclipse TS100 microscope and NIS-Elements BR 3.10 software.

### Data analysis

Data analysis for PCR was done in Excel using Student’s T-test. Analysis for differentially secreted proteins between ARCaP-E and ARCaP-M was done using Scaffold quantitative analysis T-test on normalized mass spectral counts. P-values were adjusted for multiple tests using the Benjamini-Hochberg correction. Proteins with log2 foldchange ≥2 and p-value <0.05 were considered significant.

## Supporting information

S1 Table (XLSX)

S2 Table (XLSX)

S1 Raw Images (PDF)

## List of abbreviations

ADT: Androgen deprivation therapy
ANPEP: aminopeptidases aminopeptidase N
AR: Androgen receptor
ARCaP-E/M: Androgen-repressed prostate cancer-epithelial/mesenchymal
C1S: Complement C1s subcomponent
CM: Conditioned media
ECM: Extracellular matrix
EMT: Epithelial-Mesenchymal transition
FBS: Fetal bovine serum
GO: Gene ontology
GSTP1: Glutathione S-transferase-P1
GZMB: Granzyme B
HSPG2: heparan sulfate proteoglycan core protein
KD: Knockdown
L1CAM: L1 cell adhesion molecule
LC: Liquid chromatography
LNPEP: Leucyl-cystinyl aminopeptidase
MMP: Matrix metalloproteinase
MS: Mass spectroscopy
NRCAM: neuronal cell adhesion molecule
PCR: Polymerase chain reaction
PEDF: Pigment epithelium-derived factor
ROS: Reactive oxygen species
SERPINE1: plasminogen activator inhibitor 1 gene
SERPINF1: Pigment epithelium-derived factor gene
