# Supplementary material for: Secretome analysis reveals upregulated granzyme B in human androgen-repressed prostate cancer cells with mesenchymal and invasive phenotype
Source: PLoS One. 2020 Aug 7;15(8):e0237222. doi: 10.1371/journal.pone.0237222 (PMC7413421; doi:10.1371/journal.pone.0237222)

Raw composite image for figure 2 trial 1, 2 and 3. 25ug were loaded per well for the conditioned media (CM) taken from ARCaP-E (CM-E) and ARCaP-M (CM-M).

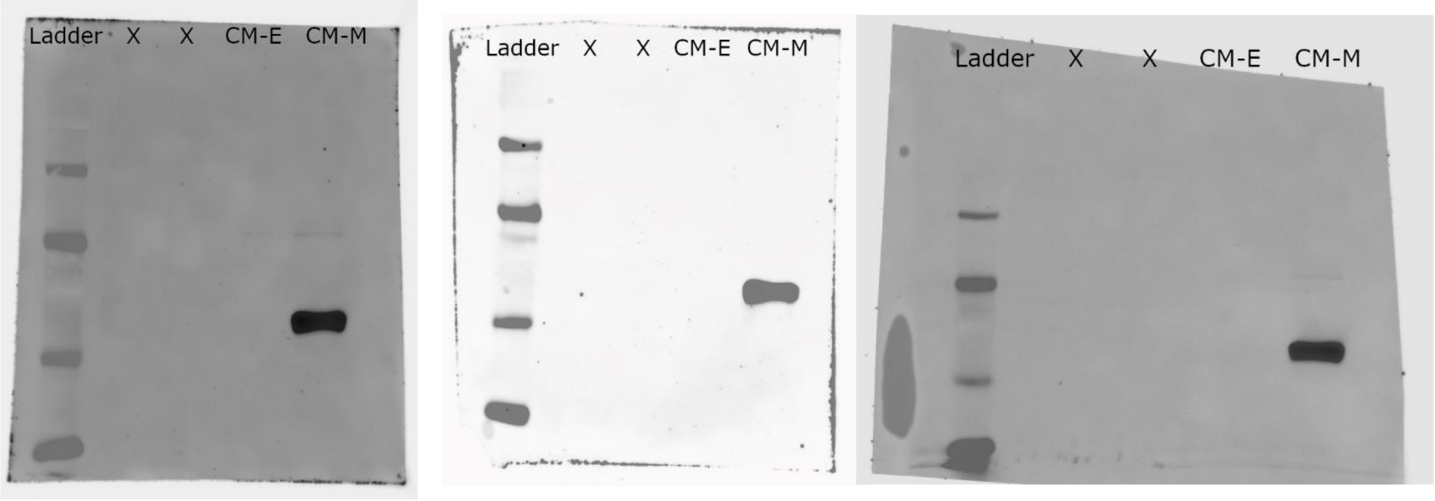

Raw composite images for figure 4 showing the control scrambled siRNA (Cntrl) and siRNA for granzyme B knock down (KD) at times 48, 72, and 96 hours

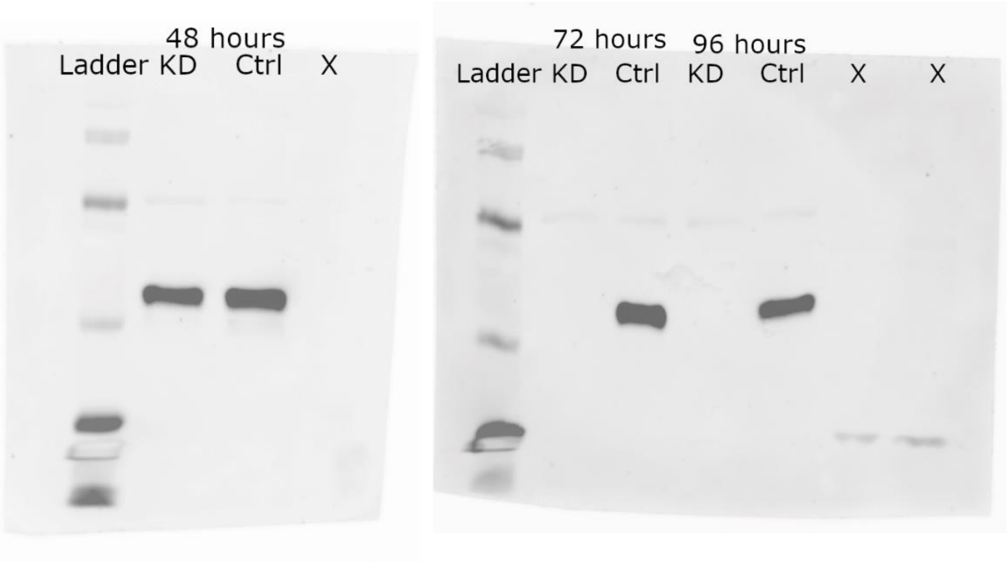

Supplement: S1 Raw Images — (PDF) [file pone.0237222.s003.pdf]
